# Effect of acute exposure of Hg on physiological parameters and transcriptome expression in silkworms (*Bombyx mori*)

**DOI:** 10.3389/fvets.2024.1405541

**Published:** 2024-06-11

**Authors:** Huanhuan Wen, Yanan Wang, Yongqiang Ji, Jing Chen, Yao Xiao, Qixiang Lu, Caiying Jiang, Qing Sheng, Zuoming Nie, Zhengying You

**Affiliations:** ^1^College of Life Sciences and Medicine, Zhejiang Sci-Tech University, Hangzhou, China; ^2^Zhejiang Provincial Key Laboratory of Silkworm Bioreactor and Biomedicine, Zhejiang Sci-Tech University, Hangzhou, China

**Keywords:** *Bombyx mori*, mercury-stress, toxicity effect, transcriptome, oxidative stress

## Abstract

Mercury (Hg) contamination poses a global threat to the environment, given its elevated ecotoxicity. Herein, we employed the lepidopteran model insect, silkworm (*Bombyx mori*), to systematically investigate the toxic effects of Hg-stress across its growth and development, histomorphology, antioxidant enzyme activities, and transcriptome responses. High doses of Hg exposure induced evident poisoning symptoms, markedly impeding the growth of silkworm larvae and escalating mortality in a dose-dependent manner. Under Hg exposure, the histomorphology of both the midgut and fat body exhibited impairments. Carboxylesterase (CarE) activity was increased in both midgut and fat body tissues responding to Hg treatment. Conversely, glutathione S-transferase (GST) levels increased in the fat body but decreased in the midgut. The transcriptomic analysis revealed that the response induced by Hg stress involved multiple metabolism processes. Significantly differently expressed genes (DEGs) exhibited strong associations with oxidative phosphorylation, nutrient metabolisms, insect hormone biosynthesis, lysosome, ribosome biogenesis in eukaryotes, and ribosome pathways in the midgut or the fat body. The findings implied that exposure to Hg might induce the oxidative stress response, attempting to compensate for impaired metabolism. Concurrently, disruptions in nutrient metabolism and insect hormone activity might hinder growth and development, leading to immune dysfunction in silkworms. These insights significantly advance our theoretical understanding of the potential mechanisms underlying Hg toxicity in invertebrate organisms.

## 1 Introduction

Mercury (Hg) is one of the highly toxic heavy metals prevalent in our environment, and its substantial accumulation via the food chain poses severe adverse effects on human health (1, 2). The historical Minamata mass epidemic in Iraq and Japan vividly showcased the profound toxicity of Hg (3, 4). Pollution by Hg stems primarily from a range of natural and anthropogenic activities, including volcanic eruptions, rock weathering, and diverse industrial processes (5). Hg exists in different forms in the environment. Elemental Hg can undergo oxidation, forming soluble species (Hg^2+^), which, through natural processes, may further convert into the highly toxic methylmercury (MeHg) species (6). Human exposure to Hg has been associated with multiple adverse effects on the gastrointestinal system, kidneys, central nervous system, and the developing fetus (7, 8). Consequently, it is imperative to thoroughly investigate the responses and mechanisms of Hg exposure on organisms.

The primary toxicity mechanism of Hg lies in its capacity to bind sulfhydryl groups in biomolecules and inhibit specific antioxidant factors, leading to the overproduction of reactive oxygen species (ROS) and triggering oxidative stress. Consequently, enzymes, lipids, and nucleic acids suffer damage, ultimately resulting in cell death (9). In response to Hg-induced oxidative stress, the induction of antioxidant defense systems becomes paramount (10). Crucial players in this defense include glutathione S-transferases (GSTs), carboxylesterases (CarEs), superoxide dismutase (SOD), catalase (CAT), cytochrome P450 monooxygenases (CYP450s), glutathione peroxidase (GPX), thioredoxin reductase (TrXR), and glutathione reductase (GR) which play pivotal roles in mitigating the adverse effects of ROS and are commonly employed as indicators of xenobiotic-mediated oxidative damage (11, 12). Another critical defense against potential environmental pollutants is associated with immune response, which poses potential threats to the host’s health, making it more susceptible to various pathogens, and elevating the risk of death from infectious diseases (13). Previous studies have suggested that the Toll and immune deficiency (IMD) signaling pathways, along with the JAK/STAT pathway and the steroid hormone 20-hydroxyecdysone (20E), play regulatory roles in the innate immunity of the silkworm (*Bombyx mori*) (14). Disruption of these signaling pathways may disturb the expression of antimicrobial peptides (AMP), resulting in immune system dysfunction. Despite the existing estimations of Hg toxicity from various perspectives, there is a scarcity of research exploring the mechanisms of damage in invertebrate animals.

Silkworm is an extensively domesticated, silk-producing, oligophagous insect primarily nourished by fresh mulberry leaves. Mulberry trees tend to accumulate heavy metal ions from soil and atmosphere, which are subsequently stored in their leaves. Silkworms feeding on these contaminated leaves exhibit diminished growth, development, and cocoon silk production, thereby posing a significant challenge to the silk industry (15). Consequently, there is a pressing need to investigate the toxicological impacts of heavy metals and elucidate detoxification mechanisms in silkworms, given the substantial environmental accumulation of heavy metals (16). Owing to its remarkable characteristics and longstanding history of artificial domestication, the silkworm has obtained considerable attention from scientists across various fields, particularly in recent years, encompassing environmental pollution, nanomaterial toxicity assessment, pesticides, and medicine (17–20). The Chinese Oak silkworm (*Antheraea pernyi*) among the best-known species of wild silkworms has been used as a well-known wild silk moth in sericultural and medicine industry for hundreds of years. Notably, they have seen increased utilization in studies involving heavy metals, with research focusing on the potential toxicity effects of cadmium (21), silver (22), uranium (23), and lead (24) *in vivo*. However, there remains a paucity of research specifically addressing Hg exposure processes, which could be pivotal in elucidating the underlying mechanisms of Hg toxicity and tracking out the vital targets for Hg tolerance.

To better understand the underlying mechanisms of Hg exposure in *B. mori*, we focused on the larval midgut and fat body. As the two pivotal organs of the silkworms, the midgut functions as the primary surface and barrier, crucial in preventing the absorption of diverse toxins into the body; the fat body, a functionally diverse tissue involved in storage, metabolism, and protein synthesis, facilitates nutrition metabolism and accommodates variable physiological demands throughout its life cycle (25, 26). Therefore, these two organs were chosen as model systems in this study to investigate the effects of Hg exposure comprehensively. In the present study, we evaluated the sensitivity of *B. mori* exposed to varying concentrations of Hg stress. Our assessment examined growth status, developmental changes, and antioxidant enzyme activities. Furthermore, we conducted a transcriptomic analysis in the midgut and fat body tissues to elucidate key genes and pathways associated with the silkworm’s response to Hg exposure. These findings aim to provide the potential toxicological effects of Hg responses at biochemical and molecular levels and offer a holistic perspective on the risk assessment of Hg exposure in the environment.

## 2 Materials and methods

### 2.1 Insect rearing and chemicals

*Nistari*, a multivoltine silkworm strain used in our experiment was sourced from the Zhejiang Academy of Agricultural Sciences (China). Silkworm larvae were reared at a temperature of 25 ± 2°C and 75% relative humidity as described in previous research (27). Larvae on the 2nd day of the 5th instar were chosen, and then randomly divided into six groups (90 larvae for each group) in each treatment, and each group was triplicated. The standard Hg liquid, containing 5% HNO_3_, was procured from Solarbio (Beijing, China).

### 2.2 Hg treatment

To examine the impact of Hg stress on silkworms, we employed five concentrations of Hg (20, 40, 60, 80, and 100 mg/L). Solutions with these Hg concentrations were used to briefly soak mulberry leaves for approximately 1 min, naturally dried, and then provided to the larvae. The mulberry leaves were dipped in deionized water and used as the untreated group. Each group was supplied with the equivalent weight of mulberry leaves. Body weight and survival rate were recorded on 1-, 2-, 3-, 4-, 5-, and 6-day treatment. After 72 h Hg exposure, silkworm midgut and fat body samples were collected from each group in triplicates. Samples were kept at −80°C for RNA extraction and enzyme activity assay.

### 2.3 Tissue processing and H and E staining

Following exposure to Hg treatment, the midgut and fat body were collected for hematoxylin-eosin (HE) staining, as described in prior studies (28, 29). Briefly, the collected samples were initially fixed in 4% paraformaldehyde at 25°C no more than 24 h, and then dehydrated using a series of gradient ethanol baths ranging from 70 to 100% to displace water, vitrified with dimethylbenzene, infiltrated by paraffin wax, and cut into sections (5 μm). The obtained tissue sections were dyed using 2% Mayer’s hematoxylin and 1% eosin. Slides were observed and the light microscope (SOPTOP, CX40) was used to capture the images.

### 2.4 Antioxidant enzyme activities assay

To detect the effects of oxidative stress under different concentrations of Hg exposure on silkworms, the enzyme activities of carboxylesterase (CarE) and glutathione S*-*transferases (GSTs) were determined after 72 h Hg exposure. The silkworm midgut and fat body samples were extracted from each group in triplicates. The homogenates of midgut and fat body in tissue lysate were centrifuged at 12,000 rpm at 4°C for 15 min. The supernatants were obtained and used for enzyme activity measurement. The BCA Protein Assay Kit (P0010, Beyotime, China) was chosen for the protein content measurement. The enzyme activities of GST and CarE were determined by glutathione S-transferase (GST) Assay Kit (Serial No: BC0355) and carboxylesterase (CarE) Assay Kit (Serial No: BC0845) from Solarbio Life Sciences (Beijing, China) by the instruction for use. At least three replications were performed for enzyme assay.

### 2.5 Total RNA preparation, library construction, and transcriptome sequencing (RNA-seq)

The total RNA of midgut and fat bodies from the group exposed with 80 mg/L Hg was obtained using TRIzol reagent (Invitrogen, Carlsbad, CA, United States) as described previously (30), and used for the transcriptome sequencing. The Hg concentration of 80 mg/L was determined by a series of preliminary experiments and relevant literature was used for the following experiments (21). Library preparation, clustering, and RNA-Seq techniques of 12 samples (with three biological replicates) were carried out at Beijing Novogene (Beijing, China). The total RNA quality and quantity were analyzed with RNA Nano 6000 Assay Kit by Agilent Technologies 2100 Bioanalyzer (Agilent Technologies, CA, United States). RIN (RNA integrity number) of the RNA samples of more than 8.5 were prepared for RNA-seq. Libraries were created with the NEB Next Ultra RNA Library Prep Kit for Illumina (NEB, United States) according to the Kit’s instructions. Using an Illumina HiSeq 2000 platform (Illumina, United States), sequencing of the 12 samples was conducted, and the paired-end reads between 125 to 150 bp were generated. After sequencing, adapter sequences, and low-quality reads were deleted, and then clean reads were generated from raw reads. GC content, Q20, and Q30 of clean reads were adopted to estimate the reliability of data. The clean data were mapped to the SilkBase (31) and KAIKObase (32) to get read information using HISAT2 (33). The expression levels of the identified genes were evaluated by fragments per kilobase of transcript per million mapped reads (FPKM) (34).

### 2.6 Bioinformatic analysis

The DEGs analysis between Hg and ultrapure water treatment was performed by DESeq2 (35). The standard of |log2FoldChange| ≥ 1.0 & *p*_adj_ ≤ 0.05 was carried out to identify the DEGs. Gene Ontology (GO) and Kyoto Encyclopedia of Genes and Genomes (KEGG) were performed for classification and pathway enrichment analysis, respectively, with *p*_adj_ ≤ 0.05.

### 2.7 Expression analysis of DEGs by quantitative real-time PCR

The real-time PCR (RT-qPCR) was performed to further confirm the reliability of RNA-Seq data. The total RNA of the midgut and fat body were extracted from the larvae treated with Hg exposure for 72 h and the control group using TRIzol reagent (Invitrogen, United States) as described above. PCR primers (Supplementary Table S1) were designed and then synthesized via Sangon Biotech (Shanghai, China). The Prime Script kit including the gDNA Eraser (Takara, China) was used to synthesize the first cDNA based on the instructions. The RT-qPCR was performed on an ABI 7500 Real-Time PCR system (Applied Biosystems, CA) using a SYBR^®^Premix Ex TaqTM II kit (Takara). The amplification reaction was carried out in a 20.0 μL reaction mixture as follows: denaturation at 95°C for 30 s followed by 40 cycles of 95°C for 5 s and 60°C for 30 s, and melting curves were constructed. The *BmRp49* (accession number: NM_001098282) was adopted as a reference and the ΔΔCt method (36) was employed to calculate the relative expression. At least three independent replicates were conducted.

### 2.8 Statistical analysis

Statistical analyses and graphs were performed using OriginPro (version 2022b). One-way ANOVA analysis (Tukey’s *post hoc* test) was used to compare variance between the experimental and control groups. The *p <* 0.05 represented the significance of the data compared to the control group.

## 3 Results

### 3.1 Impact of Hg treatment on the growth and development of *Bombyx mori*

**Figure 1:**
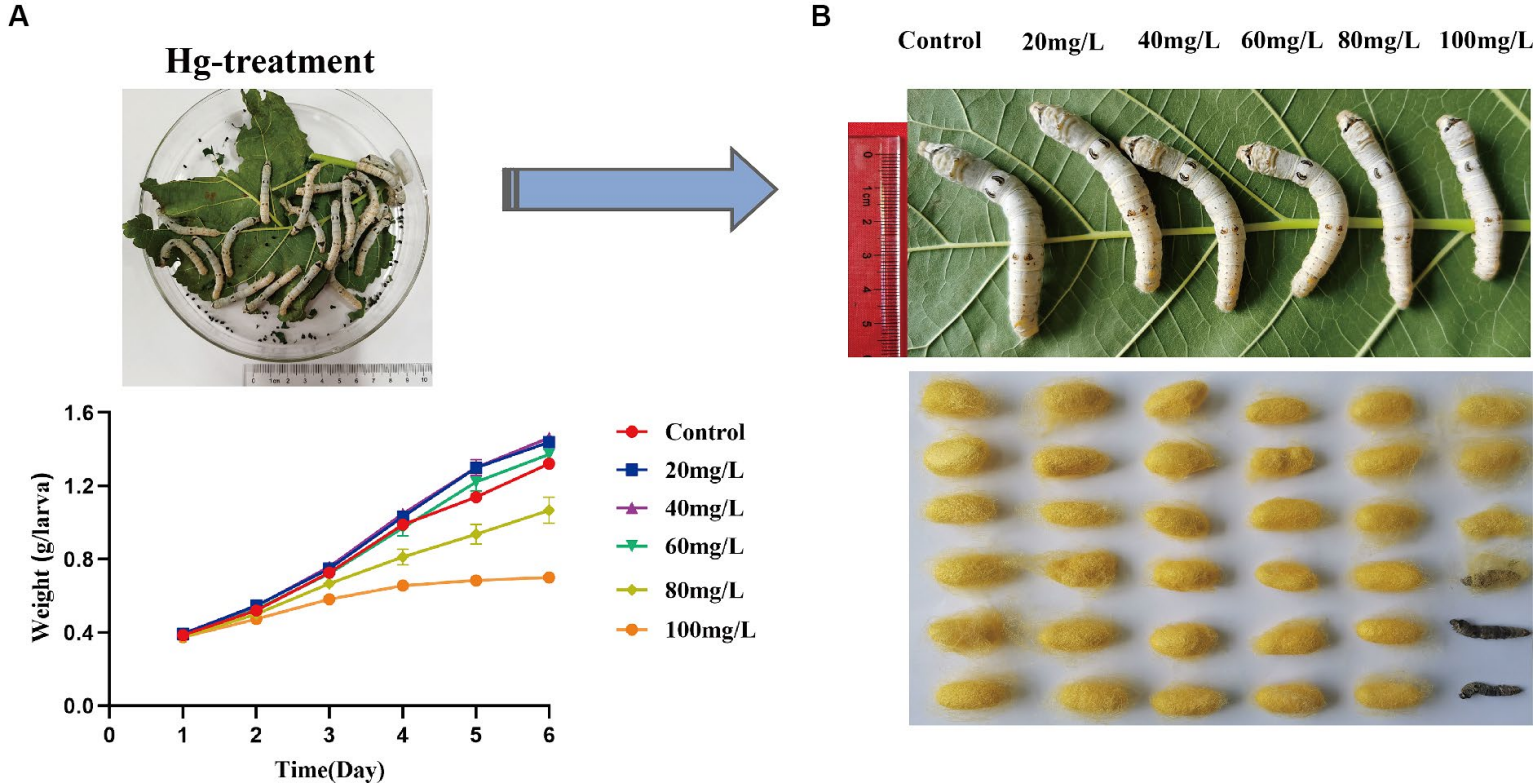
Symptoms and toxicological characteristics of silkworms caused by Hg exposure. **(A)** The trend of silkworm larvae body weight changes after 1, 2, 3, 4, 5, and 6 days of Hg exposure. **(B)** The larvae and cocoon features of 20 mg/L, 40 mg/L, 60 mg/L, 80 mg/L, and 100 mg/L Hg treatment, respectively.

**Figure 2:**
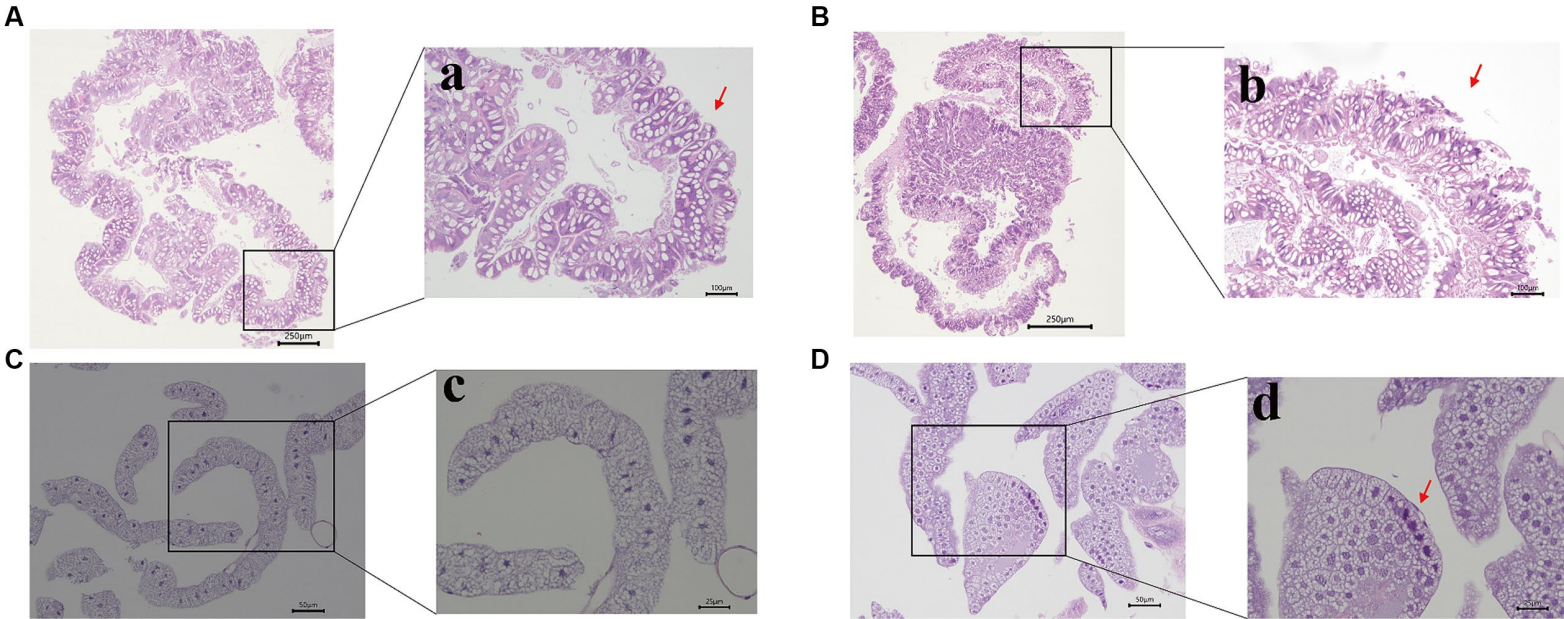
Morphological changes of the midgut and fat bodies with different treatments. **(A,B)** are the pictures of the midgut of the control and experimental group; **a,b** are pictures with high magnification. The bubble-like structure was generated in the midgut (indicated by the red arrowhead). **(C,D)** are the pictures of the fat bodies of the control and treatment group; **c,d** are pictures with high magnification. Nuclear pyknosis emerged in the fat body (indicated by the red arrowhead). Bars: **A,B**: 250 μm; **a,b**: 100 μm; **C,D**: 50 μm; **c,d**: 25 μm.

To assess the effects of Hg stress on silkworms, we exposed them to different concentrations of Hg-treated mulberry leaves for 144 h (6 days). The body weights of the low-concentration groups (20 mg/L, 40 mg/L, 60 mg/L) exhibited a slight increase compared with the control group, while the high-concentration groups (80 mg/L and 100 mg/L) experienced a reduction during Hg treatment (Figure 1A and Supplementary Table S2). Although almost all larvae successfully formed cocoons, some in the high-concentration groups, particularly at 100 mg/L, produced thinner cocoons and even succumbed (Figure 1B). Additionally, histological staining was performed to further clarify histomorphology and structure variations in midguts and fat bodies exposed to 100 mg/L Hg for 72 h. As shown in Figure 2, midgut cell morphology changed with the emergence of bubble-like structure under Hg stress. Histopathological observation of fat bodies also revealed noticeable nuclear pyknosis. These results collectively indicate that exposure to a high concentration of Hg induces morphological and structural alterations in midguts and fat bodies, significantly hindering the normal growth and development of fifth-instar silkworm larvae in a dose-dependent manner.

### 3.2 Analysis of antioxidant enzyme activity in response to Hg stress

**Figure 3:**
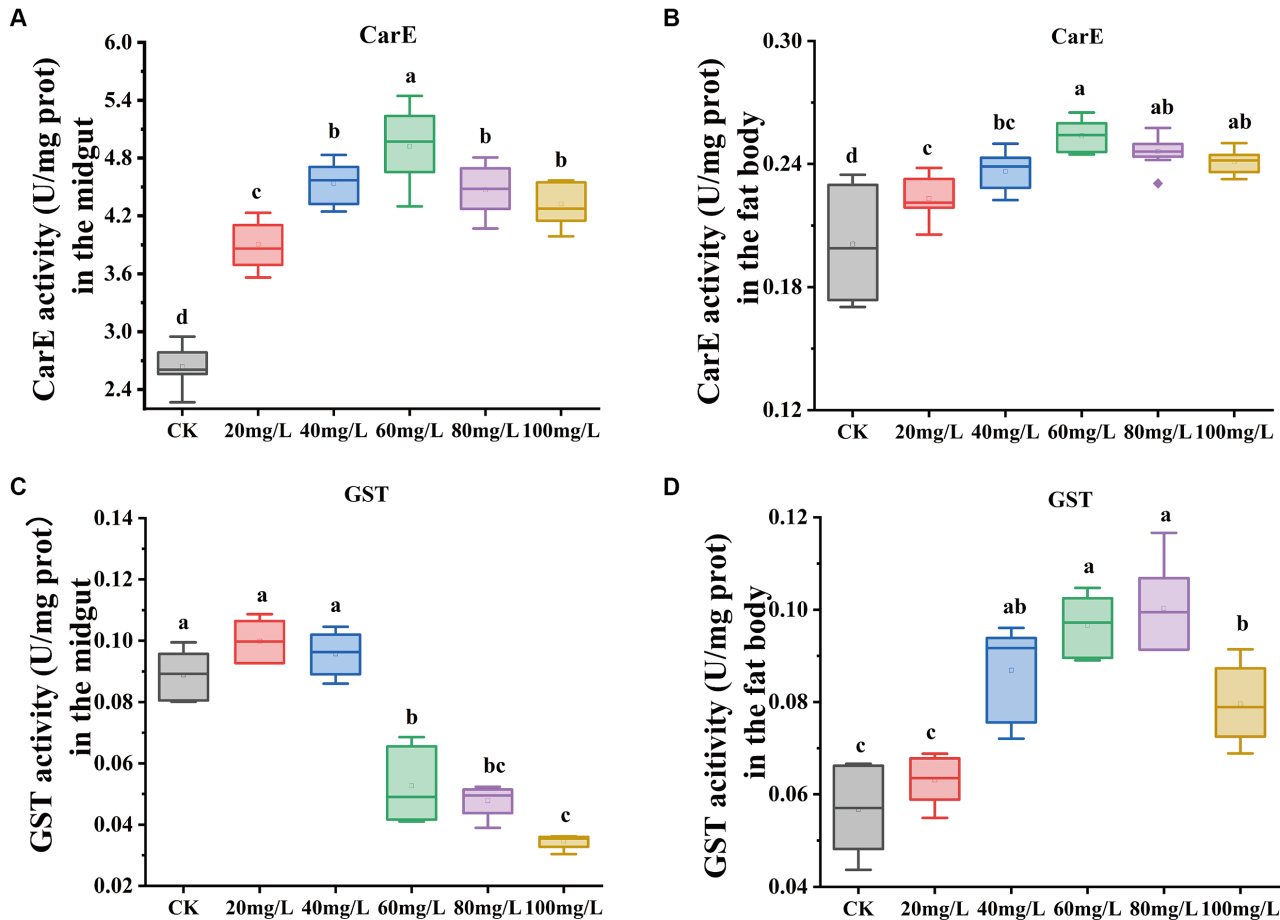
Effect on antioxidant enzyme activities in the midgut and fat body under exposure at different concentrations of Hg. **(A)** The CarE enzyme activity in the midgut. **(B)** The CarE enzyme activity in the fat body. **(C)** The GST enzyme activity in the midgut. **(D)** The GST enzyme activity in the fat body. Data are shown as the mean ± SD. Significant differences across the treatments at *p* < 0.05 were indicated using different letters (one-way ANOVA with Tukey’s *post hoc* test).

In this investigation, we observed changes in antioxidant enzyme activities between experimental and the control group after 72 h of Hg exposure. Carboxylesterase (CarE) enzyme activity exhibited a gradual increase with increased concentrations of Hg exposure both in the midgut and fat body, and then followed by a gradual decline with further concentration increase (Figures 3A,B). Conversely, Glutathione S-transferase (GST) activity in the midgut (Figure 3C) showed a significant decrease. In contrast, the fat body demonstrated a gradual increase in GST enzyme activity, and subsequently declining with further concentration (Figure 3D) increase.

### 3.3 RNA-sequencing and DEGs analysis in response to Hg exposure

**Figure 4:**
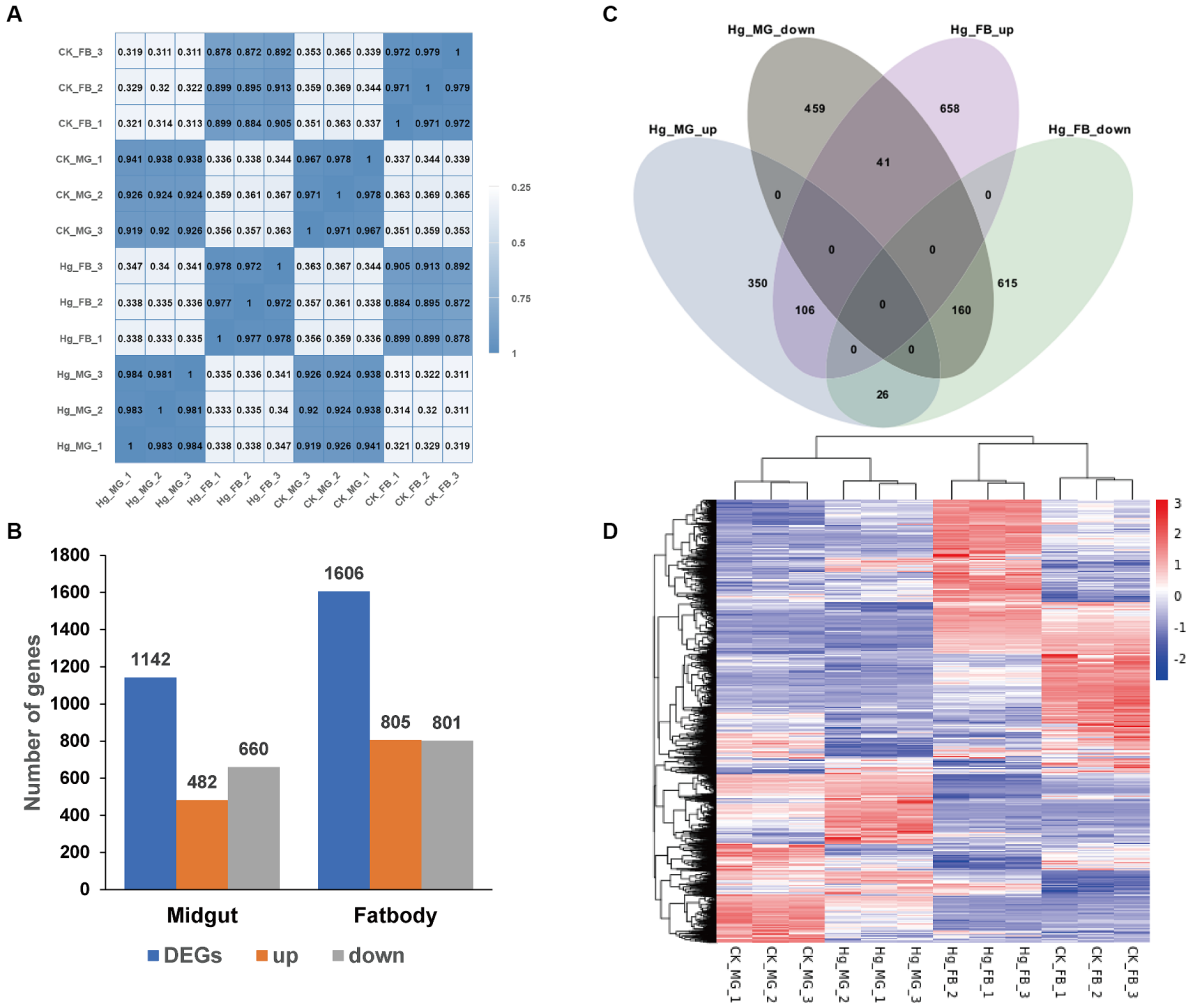
Comparative analysis of DEGs in the midgut and fat body after Hg stress. **(A)** Pearson’s correlation analysis among the 12 samples. The color of the heat map is on behalf of the degree of correlation among samples. **(B)** The overview of DEGs (the up-regulated and down-regulated genes) in the midgut and fat body. **(C)** Venn diagram analysis for DEGs in the midgut and fat body. MG, midgut; FB, fat body; up, up-regulated DEGs; down, down-regulated DEGs. **(D)** The global changes of DEGs across the 12 samples. Hierarchical clustering of DEGs was generated after Hg treatment. The color scale represents the log2 transformed FPKM value based on the row scale. Their phylogenetic relationships were shown on the left tree. The top tree showed the cluster relationship of the samples.

Data quality from transcriptome sequencing is presented in Supplementary Table S3. Following the removal of redundant and low-quality reads, each group yielded more than 40 million clean reads, representing almost 99% of the raw reads. The values of Q20 and Q30 were 97 and 93% for all quality scores, respectively, and the range of GC contents was from 44.23 to 47.55%. High consistency across different sample replicates (Figure 4A) was indicated by the Pearson correlation coefficient. Subsequently, a principal component analysis (PCA) model was established based on the expression of unigenes, revealing differences among the four groups (Supplementary Figure S1). These findings indicated that the quality of sequencing data was relatively high, and the subsequent annotation analysis could be performed.

To further assess the impact of Hg treatment on different tissues, we identified DEGs in both the midgut and fat body, comparing them with the reference groups using the DESeq2 method (Figure 4B). In the Hg_MG (80 mg/L) vs. CK_MG analysis, among the 1,142 identified DEGs, 482 were upregulated, and 660 were downregulated (Figure 4B; Supplementary Figure S2A and Supplementary Table S4). Similarly, in the analysis of Hg_FB (80 mg/L) vs. CK_FB, 805 DEGs showed increased expression, while 801 DEGs exhibited decreased expression out of the 1,606 identified DEGs (Figure 4B; Supplementary Figure S2B and Supplementary Table S5). Additionally, we found 809 and 1,273 DEGs specifically expressed in the midgut and fat body, respectively, with 333 DEGs common to both tissues, as illustrated in the Venn diagram (Figure 4C). Furthermore, for a comprehensive view of these DEGs, we conducted hierarchical clustering based on normalized FPKM values across the 12 samples (Figure 4D), offering a global understanding of gene expression changes.

### 3.4 Gene Ontology functional annotation of DEGs

**Figure 5:**
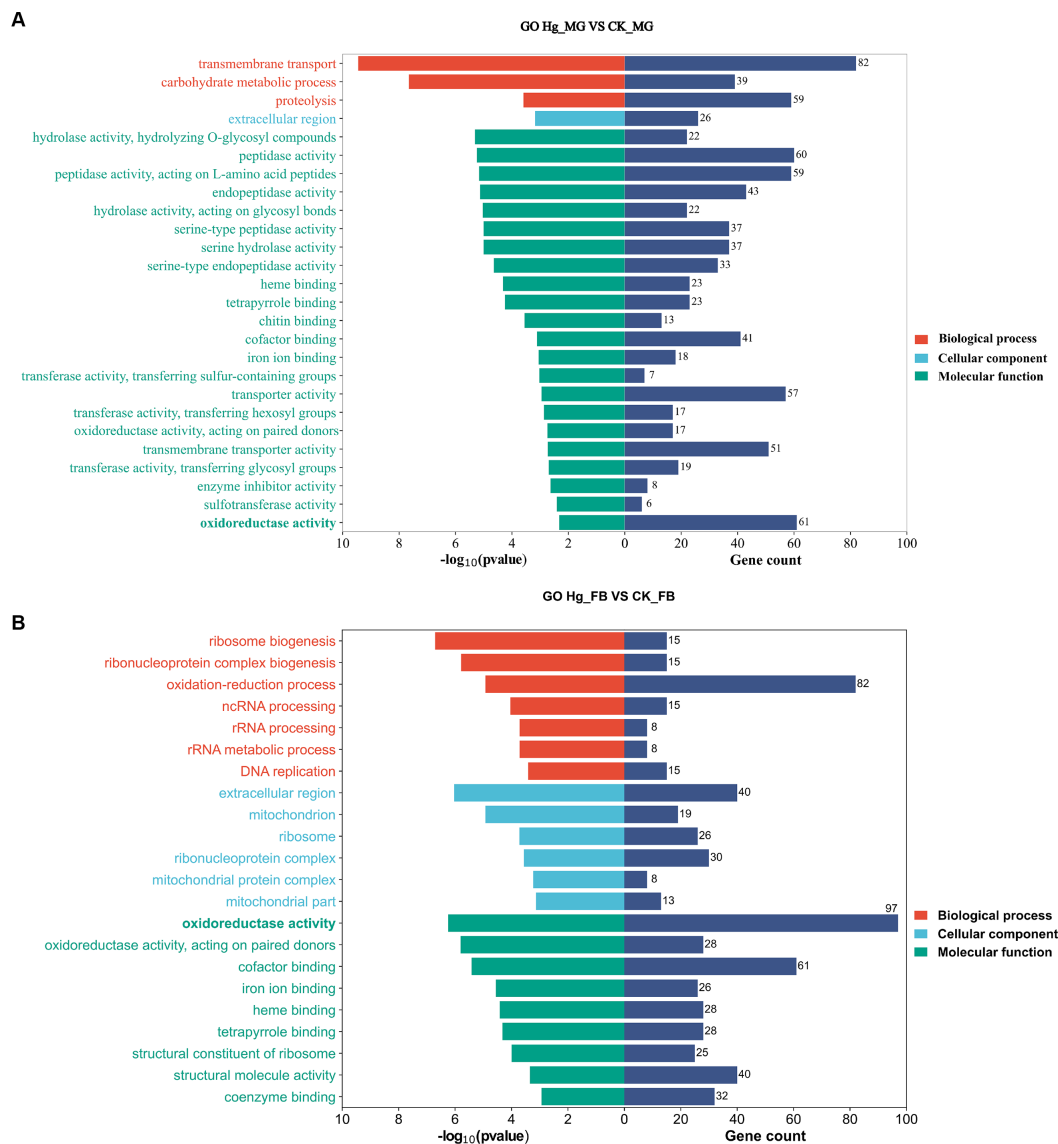
GO enrichment analysis in responsive DEGs caused by Hg stress. GO enrichment analysis of the DEGs after Hg exposure in groups **(A)** Hg_MG vs. CK_MG, and **(B)** Hg_FB vs. CK_FB. The *X*-axis is on behalf of the number of DEGs and -log_10_(*p*-value), and the *Y*-axis depicts various gene functions.

We conducted GO enrichment analysis to elucidate the correlations between DEGs and biological functions (Figure 5). In the Hg_MG vs. CK_MG group, the highest percentages of GO terms in the biological process (BP) category were associated with “transmembrane transport” (82 DEGs). Within the cellular component (CC) class, the focus was on the “extracellular region” (26 DEGs). Notably, we found that GO terms were mainly grouped into “molecular function,” with extensive assignments. The most prevalent “molecular function” assignment was “oxidoreductase activity” (61 DEGs). Additionally, crucial assignments like “peptidase activity” and “transporter activity” exhibited high enrichment levels, suggesting a widespread alteration in the molecular function of the midgut following Hg exposure (Figure 5A).

In the Hg_FB vs. CK_FB group, the most abundant category within the biological process was the “oxidation-reduction process” (82 DEGs). Cellular component categories featured prominently with DEGs distributed in the “extracellular region” (40 DEGs), “ribonucleoprotein complex” (30 DEGs), and “ribosome” (26 DEGs). Among the molecular functions, “oxidoreductase activity” (97 DEGs), “cofactor binding” (61 DEGs), and “structural molecular activity” (40 DEGs) were the predominant groups (Figure 5B). The GO analysis of DEGs in Hg_MG vs. CK_MG and Hg_MG vs. CK_MG groups are available in Supplementary Tables S6, S7, respectively. These findings underscore the involvement of numerous metabolic processes and organelle functions in response to Hg exposure in the midgut and fat body of silkworms.

### 3.5 KEGG pathway functional annotation of DEGs

**Figure 6:**
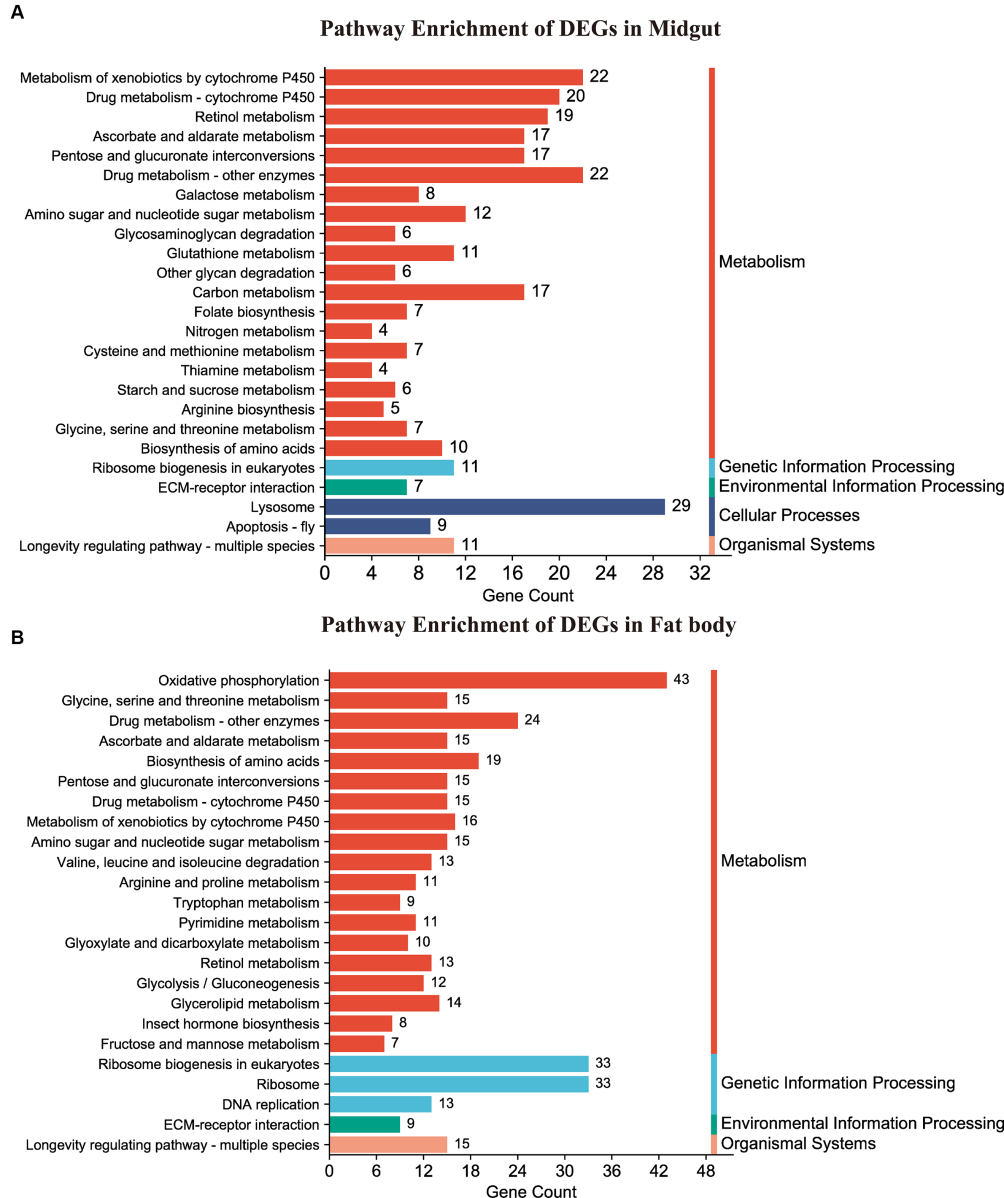
KEGG pathway enrichment analysis of DEGs. **(A,B)** Represented the DEG-enriched KEGG pathways in the midgut and fat body, respectively.

Following annotation, the enrichment analysis unveiled that a substantial number of DEGs were predominantly associated with Metabolism, Genetic information processing, Environmental information processing, Cellular processes, or Organismal systems. Notably, metabolism-related pathways exhibited a high clustering of DEGs in both the midgut and fat body organs (Figure 6). A total of 277 DEGs were allocated to 98 known pathways, with 26 pathways significantly enriched (*p* < 0.05) in the Hg_MG vs. CK_MG group, distributed across five categories (Figure 6A and Supplementary Table S8). Twenty pathways were implicated in diverse metabolic processes, with one dedicated to genetic information processing, another linked to environmental information processing, two involved in cellular processes, and one relevant to organismal systems. The highest number of DEGs was associated with the “lysosome,” followed by “metabolism of xenobiotics by cytochrome P450,” “drug metabolism-other enzymes,” and “drug metabolism-cytochrome P450,” indicating their crucial roles in the context of Hg exposure. Additionally, certain pathways demonstrated a connection between metabolism and antioxidant defense, such as “retinol metabolism,” “ascorbate and aldarate metabolism,” “carbon metabolism,” and “apoptosis-fly.” Notably, “ribosome biogenesis in eukaryotes” was linked to protein synthesis. While the “ECM-receptor interaction” pathway was associated with environmental concentrations.

In the Hg_FB vs. CK_FB group, 458 DEGs were assigned to 107 known pathways (Supplementary Table S9), which were categorized into four distinct groups. Figure 6B illustrated the 26 significant pathways (*p* < 0.05) with 19 pathways linked to metabolism, three to genetic information processing, and one to environmental information processing, one to organismal systems. As depicted in Figure 6B and detailed in Supplementary Table S9, DEGs were notably enriched in key signaling pathways, including “oxidative phosphorylation,” “ribosome biogenesis in eukaryotes,” “ribosome,” and “drug metabolism-other enzymes.” Additionally, pathways such as “biosynthesis of amino acids,” “metabolism of xenobiotics by cytochrome P450,” “drug metabolism-cytochrome P450,” “glycine, serine and threonine metabolism,” and “ascorbate and aldarate metabolism” demonstrated associations with antioxidant defense and protein metabolism.

### 3.6 Key genes involved in the insect hormone metabolism signal pathway

**Figure 7:**
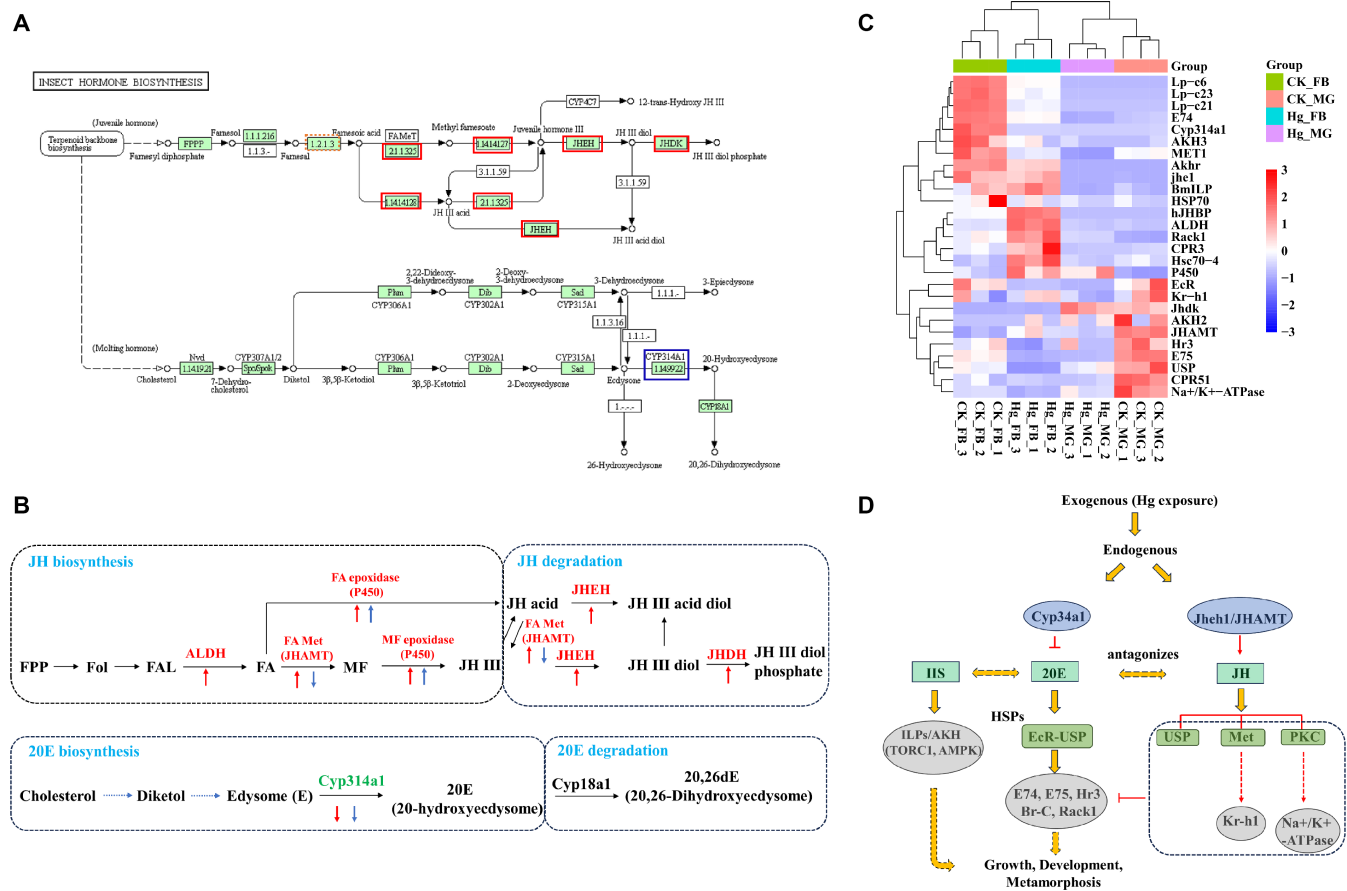
Expression pattern analysis of key genes associated with JH-20E metabolism pathway. **(A)** KEGG pathways of the insect hormone biosynthesis in the Hg_FB vs. CK_FB group. The increased DEGs were labeled with the red box, blue box represents decreased DEGs. **(B)** The DEGs are involved in the JH-20E metabolism pathway both in the midgut and fat body. The red arrow is associated with DEGs in the tissue of the fat body, the blue arrow represents DEGs in the midgut. The Up and down arrows mean up-regulated and down-regulated DEGs, respectively. **(C)** The heatmap of the important genes related to the JH-20E metabolism pathway. **(D)** Potential mechanisms of JH-20E regulated growth and development of silkworm after mercury exposure.

This investigation reveals a noteworthy down-regulation of the ecdysone oxidase gene, *Cyp314a1*, in both the midgut and fat body following exposure to Hg (Figures 7A,B). Consequently, the ecdysone receptor complex EcR/USP and several downstream transcription factor genes, including *E74*, *E75*, *Rack1*, and *Hr3*, displayed reduced expression levels. Genes associated with the juvenile hormone biosynthesis and degradation pathway, such as *ALDH*, *JHAMT*, *P450*, *JHEH*, and *JHDH*, exhibited significant upregulation in the fat body, while JHAMT experienced decreased expression in the midgut. Moreover, juvenile hormone (JH) receptor *Met* and *USP*, *Kruppel homolog 1* (*Kr-h1*), *Na^+^/K + -ATPase*, and *Lp-c* demonstrated reduced expression (Figure 7C). Additionally, alterations were observed in the expressions of *AKH2/3 and ILP*. 20-hydroxyecdysone (20E) and Insulin/insulin-like growth factor (IGF) signaling (IIS) are recognized as the two major factors regulating growth period and growth rate, respectively. These findings suggest significant perturbations in the 20E-JH biosynthesis and metabolism pathways due to Hg exposure, likely contributing to a decrease in body weight (Figure 7D).

### 3.7 RT-qPCR verification of the DEGs

**Figure 8:**
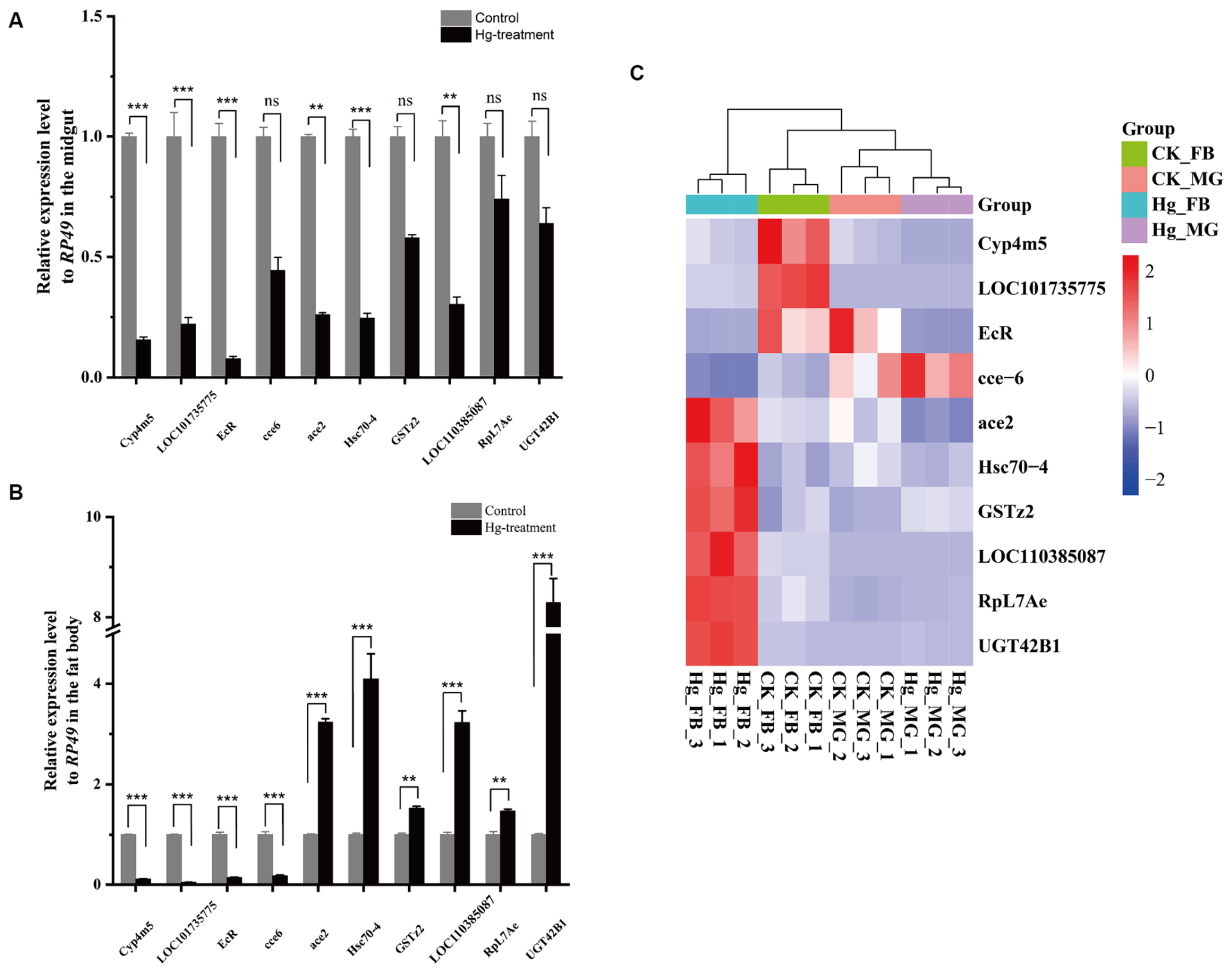
RT-qPCR analysis was carried out to confirm the DEGs identified by RNA-Seq. **(A)** The 10 DEGs in the Hg_MG vs CK_MG group were quantified by RT-qPCR. **(B)** The 10 DEGs in the Hg_FB vs CK_FB group were quantified by RT-qPCR. **(C)** The 10 DEGs in the Hg_MG vs CK_MG and Hg_FB vs CK_FB groups were quantified by RNA-seq. Data are shown as the mean ± SD of three biological replicates per treatment. The variance of DEGs between the treatment and control groups was determined by pairwise comparison (ns means no significance, ^*^p < 0.05, ^**^p < 0.01, and ^***^p < 0.001).

To assess the reliability of the RNA-seq data, we conducted an RT-qPCR experiment on a set of 10 DEGs. The expression tendency of these DEGs was highly similar to the results of RNA-Seq, demonstrating the validity of RNA-Seq data for genes with different expression levels (Figure 8 and Supplementary Table S10).

## 4 Discussion

Hg contamination poses a significant global threat to both the environment and human health, exhibiting potent neurotoxic properties and instigating a range of adverse health effects (7, 37). In this study, we investigated the detrimental effects of acute Hg exposure on silkworms under varying concentrations, examining aspects such as development, histomorphology, antioxidant enzyme activities, and transcriptome of the larval midgut and fat body. The form, dose, and duration of Hg exposure determine the toxic effects, with even trace amounts capable of causing harm in the atmosphere (8). Lower concentrations of Hg, upon acute exposure, did not induce any decline in body weight or fatality in larvae compared to the control group; in fact, there was a modest increase in body weight to a certain extent. Conversely, silkworms exposed to higher concentrations of Hg exhibited evident poisoning symptoms, significantly inhibiting their growth in a dose-dependent manner. Previous studies have noted alterations in silkworm weight following treatment with heavy metals (21, 38). In summary, the findings indicate that exposure of silkworms to high concentrations of Hg adversely influences their growth and development and interferes with fundamental metabolic processes.

Insects rely on a detoxification defense system to eliminate ROS and counteract oxidative damage (39). Essential antioxidant enzymes, including GSTs, SOD, CAT, GR, and GSHPX, serve as primary defenders against the adverse impacts of ROS and are established biomarkers for xenobiotic-mediated oxidative stress (40, 41). Our investigation revealed an up-regulation of CAT expression in the midgut, whereas a down-regulation was observed in the fat body. CAT plays a pivotal role in cellular defense against ROS damage by breaking down hydrogen peroxide (H_2_O_2_) into oxygen and water (42). Contrarily, increased GST activity was observed in the fat body, while it was inhibited in the midgut. A parallel increase in GST activity in the fat body has been documented in previous studies involving dinotefuran exposure (19), suggesting a potential association of GSTs with primary detoxification mechanisms in the fat body. Carboxylesterase (CarE) activity exhibited a gradual rise and then gradually declined with increasing concentration in both the midgut and fat body. Additionally, our results highlighted the enrichment of glutathione metabolism in the midgut. Functioning as the intracellular dominant antioxidant defense buffer, glutathione plays a pivotal role in numerous metabolic activities, sustaining intracellular glutathione homeostasis and redox balance (43). Cytochrome P450s, heme-thiolate enzymes crucial for primary defenses against various xenobiotics (44), are essential detoxification enzymes in insects, serving diverse physiological functions (45). Notably, the pathways “drug metabolism-cytochrome P450” and “metabolism of xenobiotics by cytochrome P450” were significantly enriched in both the midgut and fat body, underscoring their vital role in detoxification processes.

The DEGs were mainly involved in the poisoning process of Hg exposure, and KEGG and GO enrichment analysis identified several pivotal pathways, inducing the antioxidant defense system, the basal metabolic processes, development regulation, and immune dysfunction responses. Physiological concentrations of ROS play a crucial role in maintaining intracellular homeostasis (9). Conversely, serious oxidative stress can lead to damage to DNA, lipids, and proteins, when it exceeds the cell’s antioxidant capacity (43). Our data indicate significant enrichment of pathways related to genetic information processing, including “Ribosome biogenesis in eukaryotes” in both the midgut and fat body, and “ribosome” and “DNA replication” in the fat body. Exposure to heavy metals can induce DNA damage through ROS production, resulting in increased DNA attacks and reduced repair processes (46). Additionally, pathways associated with cellular processes such as “lysosome” and “apoptosis-fly” were enriched in the midgut. Lysosomes, acting as degradation centers and signaling hubs, play vital roles in cellular homeostasis, growth, development, and aging (47). These findings suggest a shift towards apoptotic events following Hg exposure. Therefore, elevated ROS concentrations could impede cellular proliferation and differentiation, potentially triggering apoptosis in associated cells.

Oxidoreductases constitute a diverse class of enzymes that frequently catalyze oxidation-reduction reactions, playing a crucial role in maintaining cellular redox homeostasis; aberrant expression of these enzymes can lead to the onset of various disorders (48, 49). In our study, GO analysis revealed significant enrichment of the molecular function “oxidoreductase activity” in both the midgut and fat body, while the biological process “oxidation–reduction process” was notably enriched in the fat body. Additionally, KEGG enrichment analysis of the Hg-exposed fat body versus the control fat body group demonstrated a remarkable enrichment of the “oxidative phosphorylation” pathway. Oxidative phosphorylation (OXPHOS), a central process in nearly all eukaryotic cells, occurs in the mitochondria and directly contributes to cellular adenosine triphosphate (ATP) production (50). It is well-established that mitochondria, with their highly dynamic nature, play a key role in regulating cellular homeostasis by sustaining ATP levels and generating appropriate levels of reactive oxygen species (ROS); disruption in either of these processes can lead to pathological conditions (51). Thus, these results indicate that the energy metabolism and oxidative status of silkworms were significantly affected following exposure to Hg.

Consistent with previous research, a decrease in average insect weight emerged as a common response to heavy metal treatment, attributed to elevated protein, glycogen, and lipid consumption (52), or a reduction in the anti-damage and self-repair mechanisms (21, 22, 53). In this study, the basal metabolic processes, including “amino sugar and nucleotide sugar metabolism,” “biosynthesis of amino acids,” “glycine, serine and threonine metabolism,” “pentose and glucuronate interconversions,” “ascorbate and aldarate metabolism,” and “retinol metabolism” were significantly affected both in the midgut and fat body. Additionally, alterations in “carbon metabolism” and “nitrogen metabolism” were observed specifically in the midgut, while changes in “oxidative phosphorylation,” “glycolysis/gluconeogenesis,” and “glycerolipid metabolism” were exclusive to the fat body. These changed metabolism pathways control the production, maintenance, and destruction of biomolecular, and energy balance (54). Notably, processes related to genetic information processing, such as “ribosome” and “ribosome biogenesis in eukaryotes” (55), were also enriched in the midgut or fat body following exposure to Hg. Consistent and distinct metabolic shifts were observed in both the midgut and fat body in response to Hg treatment. Carbohydrates, lipids, and proteins represent crucial energy sources for organisms, undergoing catabolism through specific biochemical cascades necessitating enzymes, cofactors, and energy (56, 57). Disruptions in these potential pathways might lead to diverse cellular disorders and metabolic diseases, which finally impact the growth and development of the larval and subsequent weight loss under high concentrations of Hg stress.

The heat shock proteins (HSPs) family plays a pivotal role in enabling organisms to shield themselves from cellular damage induced by environmental stressors, and it is crucial in regulating apoptosis and activating the innate immune system across various organisms (58). In our investigation, there was a decrease in the transcription levels of small *HSPs*, specifically *Hsp21.4*, *Hsp19.9*, *Hsp20.8*, and *Hsp20.1* in the midgut, and *Hsp19.9*, *Hsp20.8*, and *Hsp20.4* in the fat body. Within the Hsp70 family, both *Hsp70A* in the midgut and fat body, and *Hsp68* in the fat body exhibited down-regulated expressions. Notably, *Hsc70-4* demonstrated an up-regulated expression in the fat body, a phenomenon associated with *Bombyx mori* nucleopolyhedrovirus (BmNPV) infection, as reported previously (59–61). In our previous research, we observed an induction in the translational level of *BmGRP78* (also known as *Hsc70-3*) and its translocation into the cell nucleus following Hg exposure (62). These results demonstrated that these HSP proteins may be functionally related to Hg exposure. The underlying molecular mechanisms are needed to elucidate in further study.

Insects rely on the innate immune system response to defend against external invaders by producing humoral response molecules, notably antimicrobial peptides (AMPs), which can induce apoptosis or expression of antioxidant enzymes (63). Primary immune response indicators in lepidopteran insects, such as Cecropin, Attacin, and Lysozyme, were examined in the silkworm (64). Our data revealed an increase in the expression of *cecropin-D*, while *attacin1* and *enb3* exhibited decreased expression in the fat body. Additionally, *Lysozyme* showed down-regulated expression in the midgut. Exposure to Hg induced downregulated expression of *attacin1* and *enb3* in the fat body, and *Lysozyme* in the midgut, suggesting that immunotoxicity was induced in the silkworm and could be linked to the observed instances of diseased silkworms in the higher concentration exposure group. Previous studies also reported that the immunosuppressed state in the silkworms was produced by xenobiotics invaders, including insecticide (19), nanoplastics (12, 17), antibiotics (64), and silver (22). In our study, *serpin-15* exhibited up-regulated expression in the midgut, while *serpin-13*, *serpin-5*, *serpin-7*, and *serpin-3* displayed up-regulated expression, and *serpin-6* was down-regulated in the fat body. The innate immune response in invertebrate animals involves a serine protease cascade (65). Serpins, a widely distributed family of serine protease (SP) inhibitors, primarily regulate the Toll pathway, promoting the synthesis of antimicrobial peptides (AMPs) and activating the prophenoloxidase (proPO) system by inhibiting cascades of serine proteinase (66). Negative regulators such as *serpin-5*, *serpin-3*, and *serpin-15* control insect innate immunity by inhibiting clip proteinase cascades, which trigger immune responses, including the Toll pathway and melanization (67–70). In this study, the up-regulation of these serpins in the midgut or fat body suggests their potential role in the innate immune response to Hg exposure.

## 5 Conclusion

**Figure 9:**
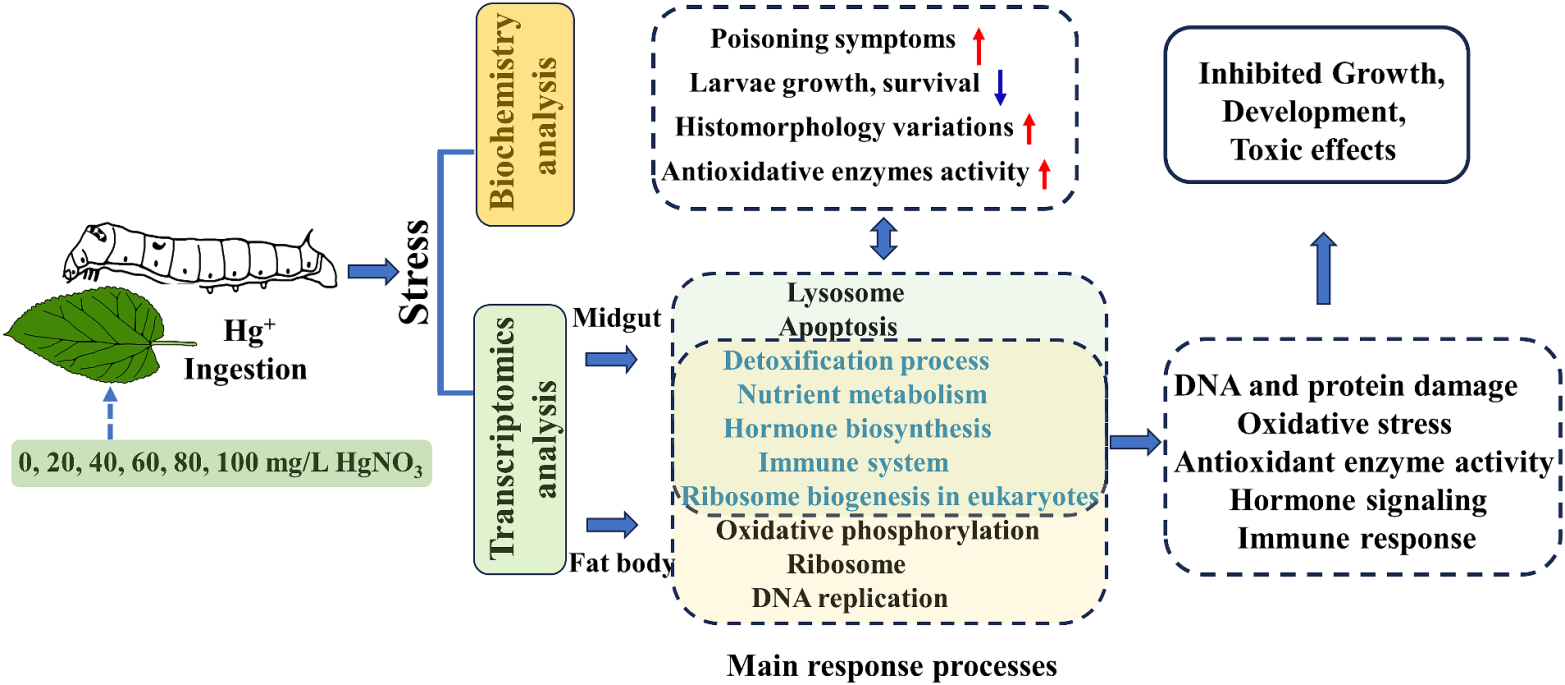
Schematic summary of Hg-stress on the biological functions of the silkworm.

In our study, the silkworms as a lepidopteran model organism were employed to assess the potential adverse effect of Hg exposure. Our results showed that higher doses of Hg exposure induced evident poisoning symptoms, decreased weight body, impaired histomorphology, and changed antioxidant enzyme activity. Transcriptomic data analysis of these tissues suggested that Hg exposure impaired fundamental metabolic processes, elicited an oxidative stress response, and induced hormone and immune dysfunction in silkworms (Figure 9). These results facilitate the knowledge of the biological effects of Hg exposure on invertebrate organisms.

## Data availability statement

The data presented in the study are deposited in the Sequence Read Archive (SRA) repository, accession number PRJNA999381.

## Ethics statement

The manuscript presents research on animals that do not require ethical approval for their study.

## Author contributions

HW: Writing – original draft, Visualization, Data curation. YW: Writing – original draft, Formal analysis, Data curation. YJ: Writing – original draft, Visualization, Investigation. JC: Writing – original draft, Software, Data curation. YX: Writing – original draft, Formal analysis, Data curation. QL: Writing – review & editing, Validation, Project administration. CJ: Writing – review & editing, Validation, Supervision. QS: Writing – review & editing, Supervision. ZN: Writing – review & editing, Supervision, Resources. ZY: Writing – review & editing, Supervision, Resources, Funding acquisition, Conceptualization.
