# Supplementary material for: Effect of acute exposure of Hg on physiological parameters and transcriptome expression in silkworms (Bombyx mori)
Source: Front Vet Sci. 2024 Jun 11;11:1405541. doi: 10.3389/fvets.2024.1405541 (PMC11196819; doi:10.3389/fvets.2024.1405541)
Supplement: Supplementary file 2 [file Data_Sheet_1.PDF]

Supplementary Material

1. Supplementary Figures

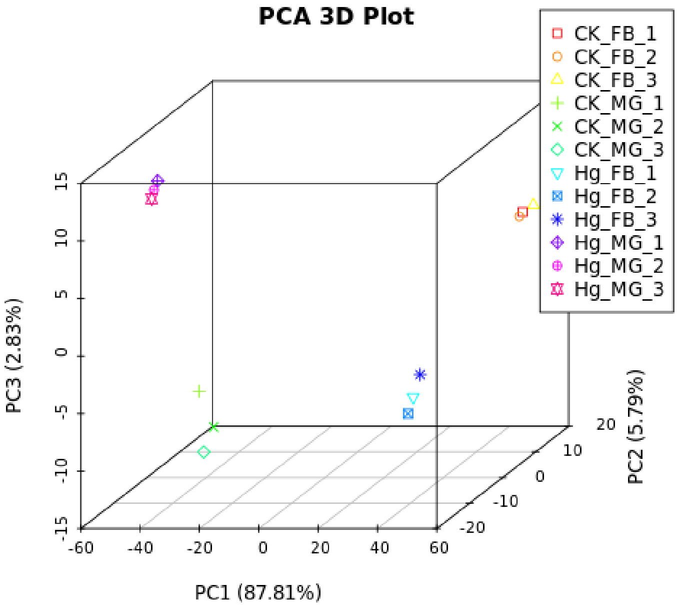

Supplementary Figure 1. PCA analysis of sequencing samples.

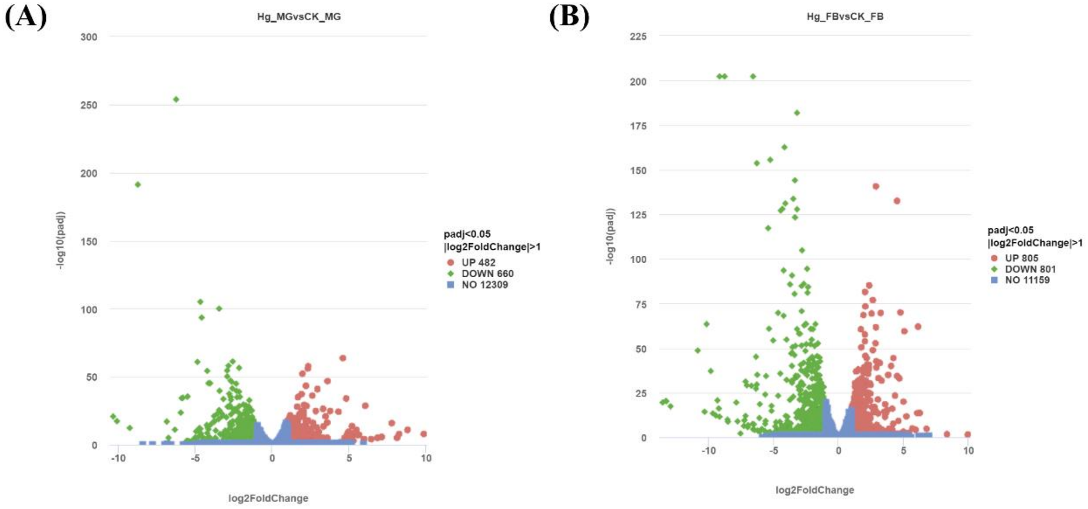

Supplementary Figure S2. Volcano plots exhibiting changes of DEGs in the midgut and fat body. Upregulation (log2FC≥1) is highlighted in red, and downregulation (log2 FC ≤-1) is highlighted in green. (A) DEGs in midgut. (B) DEGs in fat body.
